# Dynamics of price competition in Italian pharmaceutical off-patent market

**DOI:** 10.3389/fmed.2022.1045374

**Published:** 2022-11-29

**Authors:** Serena Perna, Agnese Cangini, Roberto Marini, Maria Alessandra Guerrizio, Roberto Da Cas, Giuseppe Traversa, Francesco Trotta

**Affiliations:** ^1^Agenzia Italiana del Farmaco, Rome, Italy; ^2^Istituto Superiore di Sanità, Rome, Italy

**Keywords:** drug pricing, generics, off-patent drugs, pricing competition, reference pricing system

## Abstract

**Introduction:**

The aim of the study was to evaluate, in a regulated generics market, the effect of the number of manufacturers of generic drugs on the amplitude of off-patent products price reduction and the price evolution of originators and generics after the patent expiry of pharmaceuticals dispensed by community pharmacies and reimbursed by the Italian National Health Service (INHS).

**Methods:**

The AIFA “transparency list” was utilized to select unbranded and branded off-patent drug dispensed by community pharmacies and reimbursed by the Italian National Health Service between 2012 and 2018. The unbranded drug entry in the transparency list database was considered as a proxy of its patent expiry.

**Results:**

A total of 42 different active ingredients were included in the analysis. The relative price per dose at time *t* of unbranded and branded drugs, considering as common denominator the price per dose a year before the patent expiry, (*t-1*) decreased with the increase of unbranded manufacturers. At the time of the patent expiry, the price of unbranded drugs was almost 50% less than that of branded drugs at *t-1* and the price of branded drugs started to decrease before the first unbranded entry.

**Conclusion:**

An inverse relation between the number of generic drug entrants and the price of generics and originators was detected. The patent expiry determines a price decline, more concentrated in the first year of patent expiry.

## Introduction

In a context of financial constraints, an appropriate and efficient resource allocation represents one of the main goals of health care governance at any level of health decision-making processes.

Due to their characteristics, generic medicines can play an important role in limiting the rise of pharmaceutical expenditure and in contributing to the patients’ access to high-cost innovative medicines. In fact, generic medicines are chemically and therapeutically equivalent to originator brands and are authorized to enter the market after the patent expiry of the originator. Moreover, since generic medicines are not subject to R&D (Research and Development) costs, payers can obtain prices significantly lower than the corresponding originators (1).

There are numerous policies contributing to maximize the cost-savings effect of generics, affecting both the demand and supply side. The resulting effect is the combination of the application of the different policies, both at demand and supply level (2, 3).

The main demand-side policies, which are measures that are addressed to stakeholders like physicians, pharmacists, and patients (4), encompass the followings: the prescribing by International Non-proprietary Name (INN), the generic substitution by pharmacist and the Reference Price System (5). The INN prescribing measure requires that physicians prescribe a medicine by its INN, i.e., the active ingredient; the generic substitution refers to the practice by pharmacist of substituting a medicine, whether marketed under a trade name or generic name (branded or unbranded generic), with a less expensive medicine (e.g., branded or unbranded generic), often containing the same active ingredient(s). Generic substitution may be allowed (indicative generic substitution) or required (mandatory/obligatory generic substitution). The Reference Price System (RPS), for the first time introduced in Germany in 1989 (“Festbetragssystem”), is a reimbursement policy in which interchangeable medicines are clustered into a reference group, often by the same active substance [Anatomic Therapeutic Chemical (ATC) classification level 5] or chemically related subgroup (ATC level 4). The public payer determines a price (called the “reference price”) to be reimbursed for all medicines included in the group. If the pharmacy retail price of the medicine exceeds its reference price, the patient must pay the difference, in addition to any other co-payments that may be applicable (6, 7). The supply side policies are measures that are primarily addressed to decision-makers who are responsible for the pricing and reimbursement of medicines (4) and include mainly definition of generic prices, applying generic price capping (the generic is required to be priced lower than the originator medicine and a minimum required price reduction percentage compared to the branded product can be stated).

In Box 1 policies adopted in Italy to manage and improve the uptake of off-patent medicines are summarized.

**BOX 1 Italian policies to manage and improve the uptake of off-patent medicines.**

**Demand-side**

   • Prescribing by INN

   • Generic substitution

   • Reference price system

**Supply-side**

   • Price cap

   • Specific margines

Italy has been shown to be one of the countries with the lowest degree of generic penetration and price reduction after the loss of exclusivity (8, 9).

In 2021, generic medicines represented 21.0% of the expenditure and 29.6% of community consumption. There is also a great variability across Italian regions, with generic medicines expenditure ranging from 43 to 19% of the overall off- patent medicines expenditure (mean value in 2021 was 30%). In 2021, this results in 1,1 billion euro of citizen co-payment corresponding to 18.3 euro per capita (10).

Kanavos (2) proposed five principal indicators in order to produce a methodological framework useful in estimating the performance of generic pharmaceutical policies: generic drug availability after patent expiration, time delay to generic entry, number of generic competitors, price evolution of originators and generics after loss of exclusivity and evolution of generic volume market share. In particular, the number of generic competitors measures the intensity of entry into the market post-patent expiry; therefore, it can represent a proxy of effective competition. Economic theory and cases of study confirmed that the number of competitors per molecule is associated with declining impact on prices (1, 11–15).

Several studies tried to analyze how generic medicines policies have affected price trends in European market (16–20). It was found that measures such as the price-cap and the reference pricing system, although they lead to a price reduction, may hamper the effects of generic competition.

Nevertheless, scarce evidence remains with regard to the impact of generic pharmaceutical policies, in particular in the Italian context.

The aim of the present study was to evaluate, in a regulated generics market, as Italy, how competition can disclose its effects, measuring (1) the amplitude of off-patent products price reduction according to the number of manufacturers of generic drugs and (2) the price evolution of originators and generics after the patent expiry of pharmaceuticals dispensed by community pharmacies and reimbursed by the Italian National Health Service (INHS).

## Materials and methods

### Data sources and selection

Data of the administrative database of ‘‘transparency list,’’ containing the list of reimbursable off-patent medicines and their reference prices and updated on monthly basis from AIFA were utilized to select off-patent drugs.^1^ The status of branded (ex-originator) and of unbranded drug (all drugs which have not had patent exclusivity both with generic name and with invented name) was defined according to the authorization legal basis. All medicines authorized according to the article 10 (1) directive 2001/83–“Generic Application”–Reference Product were classified as unbranded medicines.

The drugs were analyzed first according to packages level (by AIC level) and then data were aggregated at 5th level of ATC which defines the active ingredient. For each year, only drugs which had both unbranded and branded version available were included in the analysis. Moreover, to ensure stable price estimates, we selected drugs with a minimum of 10,000 packages sold for each specific year. Moreover, to take into account the average prices a year before the patent expiration, only active ingredients with index date between 2013 and 2018 were included in the analysis.

Sales data (total packages sold and total expenditure recorded by month) referred to medicines dispensed by community pharmacies and reimbursed by the INHS between 2012 and 2018, were collected through *the Medicines Utilization Monitoring Centre* (*Osservatorio Nazionale sull’impiego dei Medicinali*, OsMed) administrative database.

### Data analysis

With the aim of evaluating price reduction after the patent expiration, data were aggregated according to the time lag in years from patent expiry (*t* = −1; *t* = 0; *t* = 1; *t* = 2; *t* = 3; *t* = 4; *t* = 5). Particularly, for each active ingredient, the first date (*index date*) of generic entry in the transparency list database was considered as a proxy of its patent expiry (*t* = 0), afterward the difference of sales date from index date was calculated to consequently aggregate sales date by time lag in years from patent expiry.

For each time period considered and for each active ingredient, the average yearly price per dose, weighted considering the volume of sold boxes of single packages (i.e., single AIC), was estimated for its branded and unbranded version. Let *j* be the selected active ingredient with *n*_*m*_ the number of available different packages of the m-th market, with *m* = {*U*;*B*} unbranded and branded off-patent drugs market, respectively; let *p*_*itj*_ be the retail price per dose of i-th package belonging to the j-th active ingredient at time *t*. For each t-th year and each j-th active ingredient, the weighted average prices per dose was evaluated as follows:


$${\bar{p}}_{j\,t\,m}\,\frac{\sum_{i=1}^{n_{m}}p_{i\,t\,j}\;*\;w_{i\,t\,j}}{\sum_{i=1}^{n_{m}}\;w_{i\,t\,j}}$$

Where *w*_*itj*_ represents the total number of boxes sold of i-th package belonging to the j-th active ingredient at time *t*. Moreover, we evaluated the relative price per dose of unbranded or branded off-patent drugs at time period considered, *t*, to branded price per dose of drugs a year before the patent expiry, *t* = −1 (i.e., 12 months before the index date) or in formula: ${\bar{p}}_{j\,t\,m}/{\bar{p}}_{jB\left(t=-1\right)}$ with *m* = {*U*;*B*} unbranded and branded off-patent drugs market, respectively. The doses included in each package were determined through the defined daily dose (DDD) established by the World Health Organization Collaborating Centre (21) for Drug Statistics Methodology on the basis of the assumed average dose per day of the medicine, used for its main indication by adults.

In a first analysis, results were aggregated by competition levels, represented by the number of distinct manufacturers of unbranded off-patent drugs in the time period considered for the study.

To compare the differences among the groups, the Mann–Whitney U-test (between two groups) or Kruskal–Wallis test (between more than two groups) were used for not-normally distributed continuous variables. Multilevel mixed-effects linear regressions, to take into account multiple values of the same active ingredients during the follow-up, were applied to test, respectively: (i) the relation between the number of unbranded manufacturers on the relative price per dose at time *t* of unbranded drugs and branded drugs (Figure 2); (ii) the effect of time lag from the patent expiration on price trend of unbranded and branded drugs evaluated with separated models for unbranded and branded drugs (Supplementary Table 2). A *P*-value < 0.05 was considered statistically significant.

## Results

**FIGURE 1:**
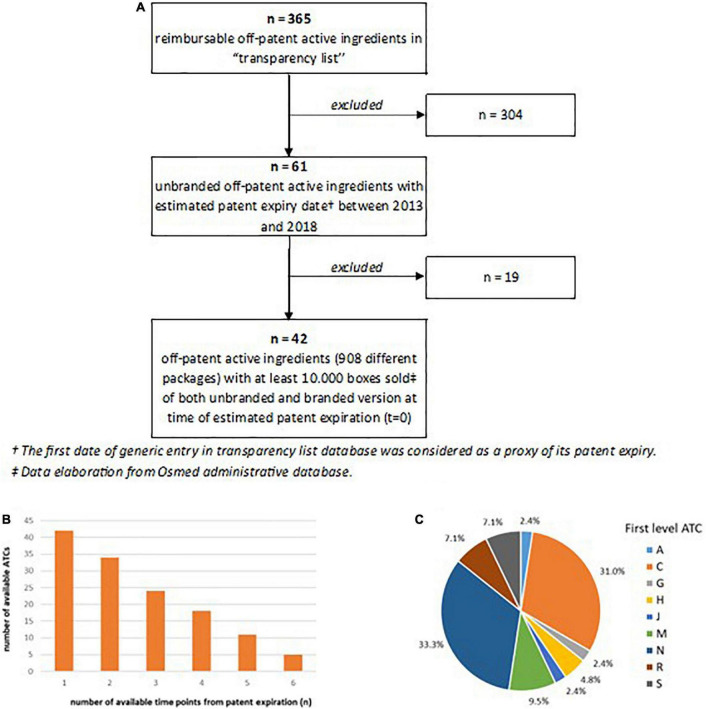
**(A)** Flowchart of selection and inclusion of medicines in the analysis (5th level ATCs); **(B)** distribution of active ingredients by number of available time point from estimated patent expiration (*t* = 0); **(C)** distribution of included active ingredients by first level ATC.

Out of the 365 active ingredients (5th level ATCs) among reimbursable off-patent medicines available in the AIFA “transparency list,” only 61 (17%) had a first date of generic entry (*index date*) in transparency list between 2013 and 2018. Among these, 42 (69%) active ingredients with a minimum of 10,000 packages of drug sold for both unbranded and branded version at time of patent expiration were included in the analysis (Figure 1A). Considering the year of first unbranded entry, the number of available time points (years) from the patent expiration was calculated. Particularly, by definition, all active ingredients have at least one available time point (*t* = 0), while only 18 (43%) have four available time points (*t* = 0–3) and only 5 active ingredients (12%) have six available time points (*t* = 0–5) (Figure 1B). The most frequent first levels of ATC observed among the included active ingredients were antiinfectives for systemic use substances (33%), followed by cardiovascular system substances (31%) and musculo-skeletal system substances (10%) (Figure 1C). A detailed description of the included active ingredients together with the year of first unbranded entry in transparency list was reported in Supplementary Table 1.

**FIGURE 2:**
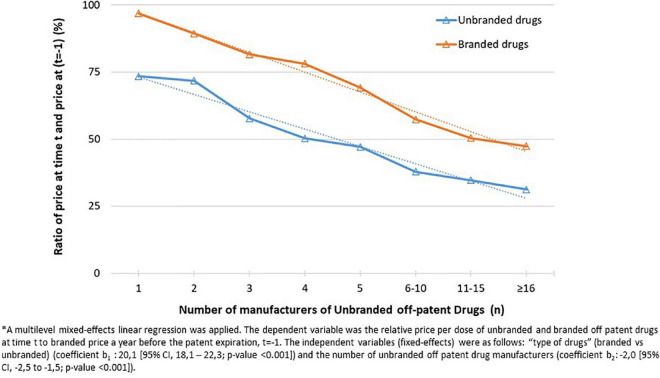
Relative price per dose of unbranded and branded off patent drugs at time t (*t* = 2013, 2018) to branded price a year before the patent expiration, *t* = –1 (i.e., 12 months before the index date) aggregated by number of unbranded off patent drug manufacturers.

The relation between the number of unbranded manufacturers and total market sales was investigated. As expected, the median number of unbranded producers increased as total market sales become larger (Supplementary Figure 1). On average, as the number of unbranded manufacturers increased, both the relative price per dose at time *t* (*t* = 2013, 2018), of unbranded drugs and branded drugs considering as common denominator the price per dose of the medicines a year before the patent expiry (*t*-1) decreased (from 97 to 47% for branded drugs and from 73 to 31% for unbranded drugs; *p* < 0,001) (Figure 2).

**FIGURE 3:**
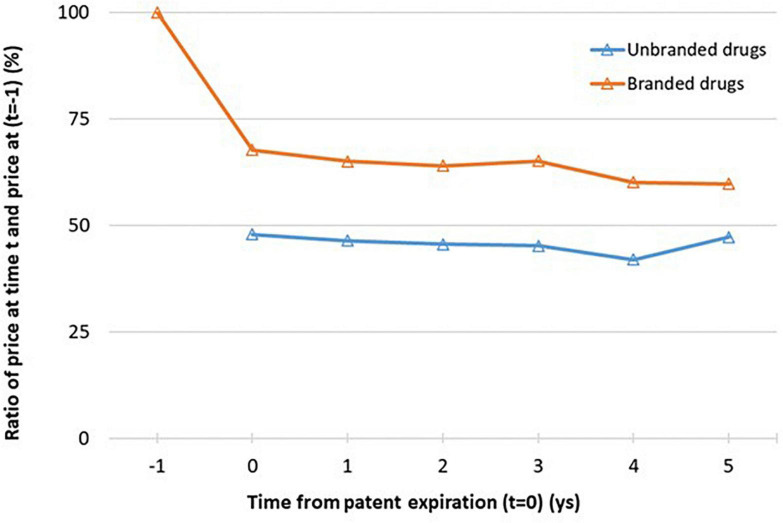
Relative price per dose of unbranded and branded drugs at lag time t (*t* = 0,…,6) to branded price a year before the patent expiration, *t* = −1 (i.e. 12 months before the index date). See Figure 1b for the number of available ATCs for each time point. *Sample size at each time point: n = 42 (*t* = 0), n = 34 (*t* = 1), n = 24 (*t* = 2), n = 18 (*t* = 3), n = 11 (*t* = 4), n = 5 (*t* = 5).

In Figure 3 and Supplementary Table 2 we analyzed the price trend by time lag from the patent expiration. At the time of the medicine entry in transparency list (*t* = 0), the weighted average price per dose of unbranded drugs was almost 50% less than the weighted average price per dose of branded drugs a year before the patent expiry (*t*-1), fixed denominator at each time point. The weighted average price per dose of branded drugs at *t* = 0, time of first unbranded entry, was almost 32% less than the weighted average price per dose of branded drugs a year before the patent expiry (*t*-1) (*p* < 0,001). Moreover, as expected at the year of first unbranded entry, the weighted average price per dose of unbranded drugs was almost 20% less than the weighted average price per dose of branded drugs. After *t* = 0, weighted average price per dose of unbranded and branded drugs remained almost constant, only a slight reduction was observed as confirmed by statistical results [from 68 to 60% for branded drugs and from 48 to 42% for unbranded drugs excluding the last time point (*t* = 5) evaluated on a very small sample size] (Figure 3). Considering the 5 years after the entry of first unbranded medicine in transparency list, the average price reduction was about 40% for branded and about 58% for unbranded price in comparison to the medicine price a year before the patent expiry (*t*−1).

More specifically, selecting only the ATCs with at least 4 time points (*t* = 0−3) the same trend was observed and the relationship within relative price per dose and the weighted average price per dose is shown more clearly (Supplementary Figure 2). The trends analysis over time from patent expiration of specific ATCs, selected firstly on the basis of year of first medicine entry in transparency list (2013 or 2016) and secondly on total amount of market sales, were also reported (Supplementary Figure 3). Almost for all molecules the same trend of weighted average price per dose of unbranded and branded was observed; at the time of first unbranded entry in transparency list the weighted average price per dose of branded drug dropped to stabilize at a constant difference with the weighted average price per dose of branded drug. Moreover, when more years before patent expiration were available, it can be seen that the weighted average price per dose of branded drug was almost constant. Pregabalin represented a particular case, since the originator has shown a price reduction only after 2 years from the generic entry into the market. It could be due to the fact that the first patent expiration referred to two of the three authorized therapeutic indications and only after 2 years the patent has expired for the third indication in the neuropathic pain.

**FIGURE 4:**
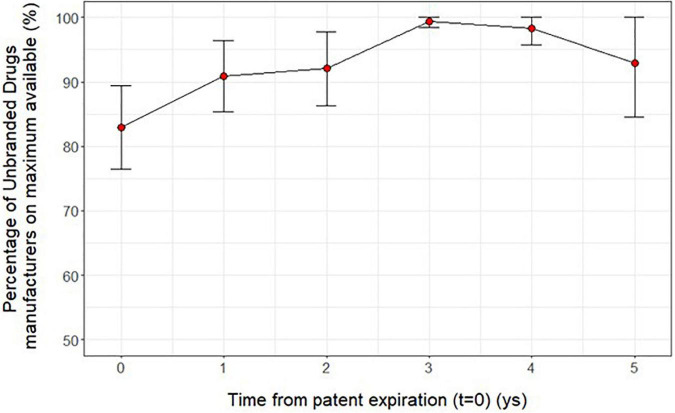
Average ratio (mean and 95 % CI) between the number of unbranded manufacturers over the maximum number observed for each active ingredient (5th level ATC) by time from patent expiration (*t* = 0−5).

**FIGURE 5:**
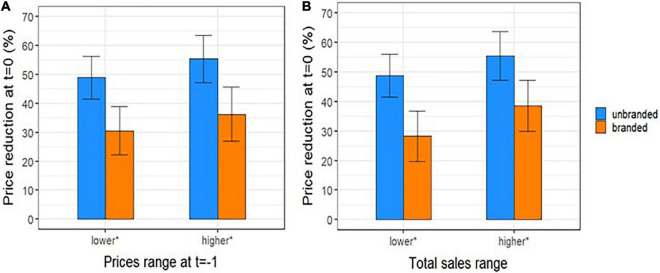
Percentage reduction (mean and 95 % CI) between price per dose of unbranded and branded off-patent drugs at lag time *t* (*t* = 0) and branded price a year before the patent expiration, *t* = –1 (i.e., 12 months before the index date) by **(A)** category of price at time *t* = –1 and **(B)** by total sales range at *t* = 0. *Median values (1,02 € and 19,6 million of €) were considered as cut-off to categorize prices per dose at *t* = –1 and total sales at *t* = 0, respectively.

Trying to investigate more deeply the results previously found, we observed that at time *t* = 0 the percentage ratio between of the number of unbranded manufacturers over the maximum number observed for each ATC is around 83%; this ratio slightly increased by time from patent expiration (*t* = 0−5) (Figure 4). Moreover, the price reductions of unbranded and branded drugs between a year before the patent expiry (*t*-1) and the time of unbranded entry (*t* = 0) were slightly higher by the increasing of the price at time *t* = −1 and total sales range although without a statistical significance (Figure 5). In fact, the price reduction between higher and lower price groups of drugs varied from 48.9 to 55.4% (*p* = 0.162) and from 35.6 to 29.1% (*p* = 0.343) for branded and unbranded drugs, respectively. Instead, the price reduction between higher and lower total sales range groups of drugs varied from 48.8 to 55.5% (*p* = 0.184) and from 28.3 to 38.5% (*p* = 0.055) for branded and unbranded drugs, respectively.

## Discussion

This study tried to investigate the dynamics of off-patent products prices after the patent expiry and to explore the effect of generic competition on prices of Italian off-patent market of pharmaceutical dispensed by community pharmacies and reimbursed by the INHS.

Our results have shown the inverse relation between price and number of generic manufacturers: we observed that in comparison to the drug prices a year before the first generic drug entry in the transparency list, both unbranded and branded products prices decreased with the increase of number of generic manufacturers with a larger variation for branded products. Differently in other countries (mainly USA and France) the price of originator has been found rigid or may even can increase after the patent expiration (22). Moreover, we investigated the price trend along time period from the expiration date. We observed that already at the time of the medicine entry in transparency list (*t* = 0), the price per dose of unbranded drugs was almost 50% less than the weighted average price of branded drugs a year before the patent expiry (*t*-1) and the price per dose of branded drugs started to decrease before the first generic entry. It is explained by the application of the reference price system according to which the patient is required to pay the difference between the reference price and the price of the purchased product. The manufacturer is encouraged to reduce the price in order to minimize the citizen co-payment and to maintain market shares (23, 24). Indeed, the study highlighted that the price reductions of generic and non-generic drugs between a year before the patent expiry (*t*-1) and the time of generic entry (*t* = 0) were slightly more intense by the increasing of the price at time *t* = −1, although a statistical significance was not found.

As a result of the reference-price system, unbranded products use does not increase if the originator price decreases to the level of the reference price (2).

After *t* = 0, the prices maintain a stable trend with a constant difference between the unbranded and branded products. The price per dose of unbranded drugs was almost 20% less than the price of branded drugs at time 0 coherently with the Italian generic pricing policy, according to that generic products have to be priced at least 20% lower than their comparable originator products in order to be granted the reimbursement. These findings were coherent with results in Kanavos (2) which found that 12 months post-patent expiry, a 16% price decline for generics was found, increasing to 21% in 24 months post-patent expiry.

The findings of our study showed that the effect of market competition is concentrated mainly at the time of transparency list entry when the majority of generic products makes entry into the market.

Differently in an unregulated market, as USA, (25) it was found that prices continue to decrease following 3 years after generics entry.

Therefore, in light of our results some policies can be suggested to promote the generics’ producers entry into the market after the first year of patent expiration; by fostering the generic drugs uptake (e.g., promoting information among patients and physicians) to make the off-patent market more attractive for unbranded medicines producers. Indeed, literature suggested that high generic market share countries could see a larger decrease in medicine prices than low market share countries (26); by simplifying the pricing and reimbursement procedures for generics; in October 2020 the Italian Medicine Agency (AIFA) has issued a new simplified procedure for pricing and reimbursement of generic products. In the case that the Manufacturer presents a price proposal taking into account discount classes pre-defined on the basis of the molecule NHS spending in the last 3 years, the product will undergo a simplified procedure for pricing and reimbursement. This procedure aims at reducing the time for generic products entry into the market and consequently at speeding up the savings realization for the INHS.

## Strengths and limitations

The present study has not only confirmed the inverse relation between price and number of generic manufacturers, shown in other studies retrievable in literature, but the authors of this work has tried to study more deeply the price dynamics into the Italian market, assessing them along the time: in the year before the generics’ entry and in the following years. Moreover, the study has analyzed the distribution of the number of manufacturers along the time after the patent expiration. Differently from other studies, the present work did not refer to a single therapeutic area but to different drugs categories. Another point to highlight is that analyzed data were related to medicines dispensed in the community on the whole national territory. The Italian off-patent drug market provides a useful example of a regulated system suggesting useful implications for policies transferable to other similar contexts. The results of this study can be useful to understand the pharmaceutical price developments and possible savings for INHS deriving from the generic drugs entry into the market. Nevertheless, the study presents some limitations: the first limitation refers to how we have extrapolated the date of patent expiry, as the date of first generic entry in the in the Transparency List. Second, it could be highlighted that the study period is short and maybe not sufficient to explore the price dynamics competition. Moreover, the study has not evaluated the effect of patent expiry on the consumption of on-patent competitors. Finally, since the sales data refer to medicines dispensed by community pharmacies, medicines being subject to tender procedures were not included in the analysis.

## Conclusion

The study detected inverse relation between the number of generic entrants and the price of generics and originators. Non-generics prices fell down more rapidly than generics. This study demonstrated that the patent expiry determines a price decline both for generics and originators, following the same decreasing trend and leaving almost constant the difference among the two groups. The price decline and manufacturer entry were concentrated in the first year of patent expiry.

## Data availability statement

The data that support the findings of this study are available on request from the corresponding author. Requests to access these datasets should be directed to AC, a.cangini@aifa.gov.it.

## Author contributions

AC contributed to the conception and the design of the work and the interpretation of data, and drafted the manuscript. SP contributed to conception and the design of the work, the acquisition of data, the analysis, and drafted the manuscript. RM and MAG contributed to the acquisition of data and the analysis and the revision of the draft. RDC contributed to the design of the work and the revision of the work draft. GT contributed to the design of the work, the interpretation of data, and the revision of the work draft. FT contributed to the conception and design of the work, the acquisition of data, the interpretation of data, and the revision of the work draft. All authors contributed to the article and approved the submitted version.

## Footnotes

1
https://www.aifa.gov.it/liste-di-trasparenza
