# Supplementary material for: Dynamics of price competition in Italian pharmaceutical off-patent market
Source: Front Med (Lausanne). 2022 Nov 29;9:1045374. doi: 10.3389/fmed.2022.1045374 (PMC9744788; doi:10.3389/fmed.2022.1045374)

# SUPPLEMENTARY MATERIAL

**Title: Dynamic of price competition of Italian pharmaceuticals off-patent markets.**

# Tables and Figures

### **Table S1**. List of active ingredients included in the analysis.

| **Active ingredient** | **ATC** | **Year of Patent Expirations (t=0)** | **N. of available packages at t=0** |
| --- | --- | --- | --- |
| acarbose | A10BF01 | 2013 | 6 |
| ivabradine | C01EB17 | 2018 | 26 |
| eplerenone | C03DA04 | 2015 | 8 |
| zofenopril and diuretics | C09BA15 | 2016 | 4 |
| enalapril and lercanidipine | C09BB02 | 2017 | 8 |
| telmisartasn | C09CA07 | 2013 | 39 |
| olmesartan medoxomil | C09CA08 | 2017 | 39 |
| telmisartan and diuretics | C09DA07 | 2014 | 32 |
| olmesartan medoxomil and diuretics | C09DA08 | 2017 | 42 |
| olmesartan medoxomil and amlodipine | C09DB02 | 2018 | 19 |
| rosuvastatin | C10AA07 | 2018 | 106 |
| omega-3-triglycerides incl. other esters and acids | C10AX06 | 2014 | 6 |
| ezetimibe | C10AX09 | 2018 | 16 |
| simvastatin and ezetimibe | C10BA02 | 2018 | 24 |
| dutasteride | G04CB02 | 2017 | 16 |
| prednisone | H02AB07 | 2015 | 16 |
| levothyroxine sodium | H03AA01 | 2013 | 18 |
| moxifloxacin | J01MA14 | 2014 | 4 |
| aceclofenac | M01AB16 | 2015 | 4 |
| celecoxib | M01AH01 | 2014 | 13 |
| eteroxib | M01AH05 | 2017 | 39 |
| alendronic acid and colecalciferol | M05BB03 | 2017 | 20 |
| rizatriptan | N02CC04 | 2013 | 28 |
| almotriptan | N02CC05 | 2015 | 10 |
| eletriptan | N02CC06 | 2016 | 7 |
| frovatriptan | N02CC07 | 2015 | 10 |
| oxcarbazepine | N03AF02 | 2015 | 4 |
| zonisamide | N03AX15 | 2016 | 12 |
| pregabalin | N03AX16 | 2015 | 65 |
| levodopa, decarboxylase inhibitor and comt inhibitor | N04BA03 | 2016 | 28 |
| rasagiline | N04BD02 | 2016 | 11 |
| aripiprazole | N05AX12 | 2016 | 32 |
| escitalopram | N06AB10 | 2014 | 55 |
| bupropion | N06AX12 | 2017 | 3 |
| duloxetine | N06AX21 | 2015 | 34 |
| memantine | N06DX01 | 2013 | 13 |
| beclometasone | R03BA01 | 2018 | 7 |
| montelukast | R03DC03 | 2013 | 58 |
| rupatadine | R06AX28 | 2017 | 7 |
| brinzolamide | S01EC04 | 2015 | 6 |
| bimatoprost | S01EE03 | 2018 | 2 |
| travoprost | S01EE04 | 2017 | 11 |

### **Table S2** - Parameter estimates of multilevel mixed-effects linear regression analysis to study the effect of time lag from the patent expiration on price trend of unbranded and branded drugs.

| **Model** | **Value†** | **Std. Error** | **p-value** |
| --- | --- | --- | --- |
| Model 1: Unbranded drugs† |  |  |  |
| *1 (Baseline)* |  |  |  |
| *0* | 1.03 | 0.36 | **0.005** |
| *2* | -0.42 | 0.41 | 0.311 |
| *3* | -0.46 | 0.45 | 0.313 |
| *4* | -1.04 | 0.55 | 0.062 |
| *5* | -0.90 | 0.76 | 0.243 |
| Model 2: Branded drugs† |  |  |  |
| *0 (Baseline)* |  |  |  |
| *-1.0* | 32.3 | 2.417 | **<0.001** |
| *1.0* | -3.1 | 2.606 | 0.242 |
| *2.0* | -4.4 | 2.952 | 0.138 |
| *3.0* | -4.2 | 3.285 | 0.205 |
| *4.0* | -5.0 | 3.988 | 0.213 |
| *5.0* | -5.3 | 5.596 | 0.346 |
| † The dependent variable (Y) was the relative price per dose of unbranded (Model 1 ) or branded (Model 2) drugs to price a year before the patent expiration, t=-1. The independent variable (fixed-effect) was time lag from the patent expiration inserted in the models as category variable to explore the average difference of Y between each time lag in comparison to baseline. The baseline category was chosen after observing exploratory analysis (Figure 3). Coefficient value of mixed-effect linear regression, its standard error and its p-value were reported. | | | |

### **Figure S1**. Relationship between total market sales volume and number of unbranded off-patent drugs manufacturers. Total market sales volume (millions of €) was categorized through statistics quartiles (first category: market sales ≤ 9.5 (first quartile); second category: 9.5 < market sales ≤ 28.6 ; third category 28.6 < market sales ≤ 44.2 (third quartile); fourth category: market sales > 44.2 .


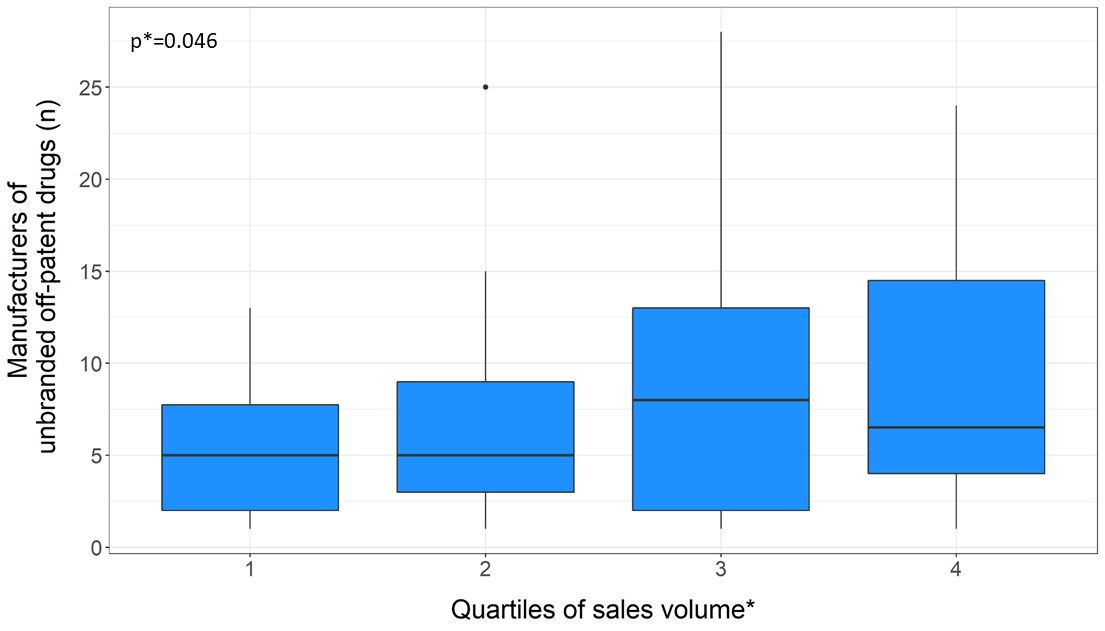


*Non parametic Kruskal–Wallis test was used to compare mean among four groups.

### **Figure S2.** (a) Relative price per dose of unbranded and branded off-patent drugs at lag time t (t=0, …, 6) to branded price at patent expiry (t=0); b) weighted average yearly prices per dose of unbranded and branded off-patent drugs at lag time t (t=0, …, 6) from patent expiry(t=0). Selection of medicines (5^th^ level ATC) with complete information on four time points from patent expiration (n=18).

a)


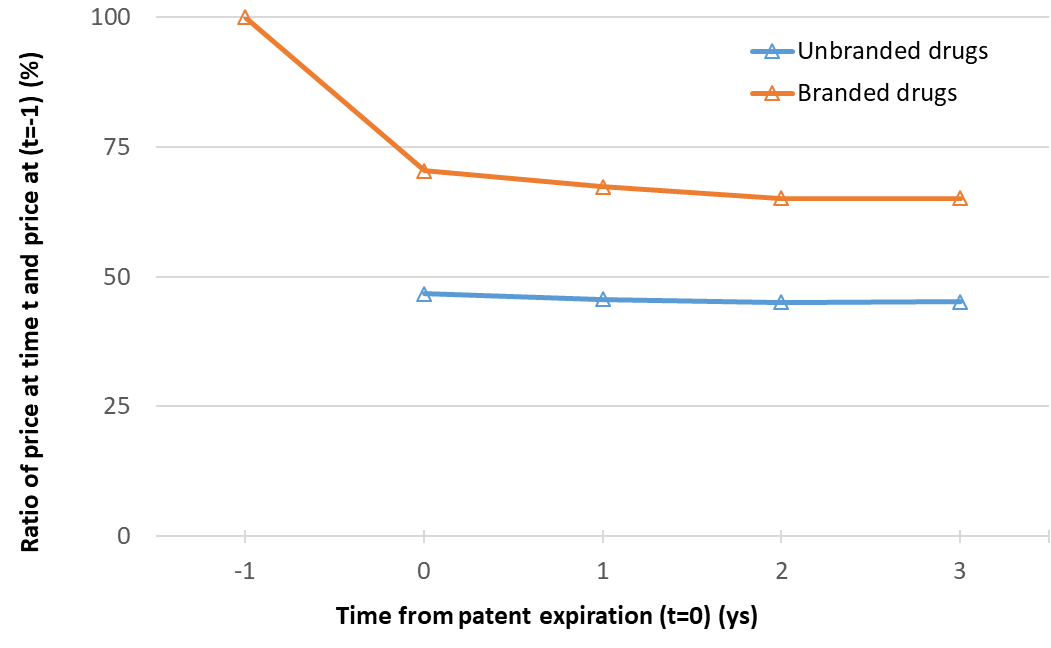


b)


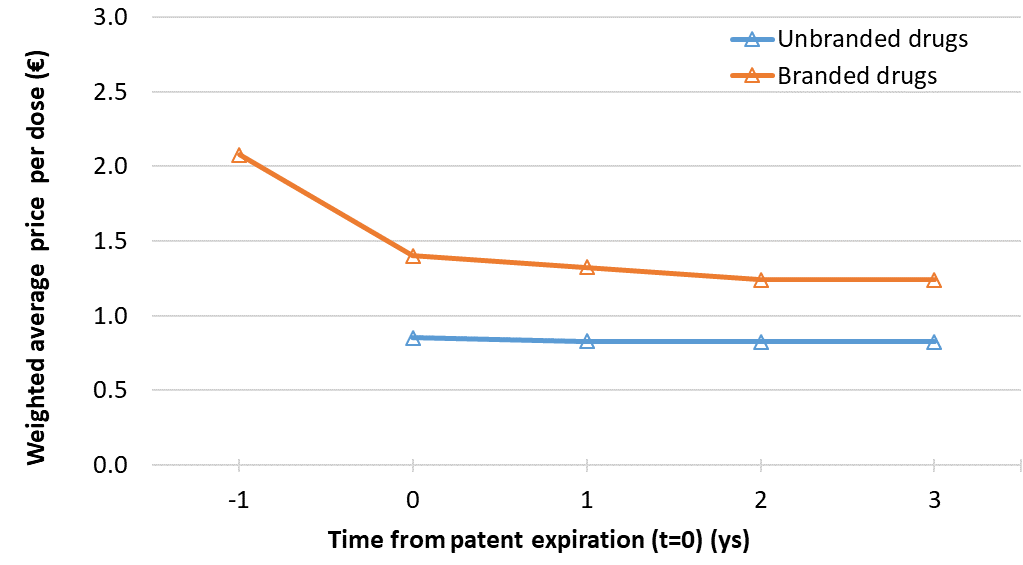


### **Figure S3**. Weighted average yearly prices per dose of unbranded and branded off-patent drugs at lag time t (t=0, …, 6) for specific medicine (5^th^ level ATC) grouped by year of patent expiration.

**a) year of patent expiration = 2013**


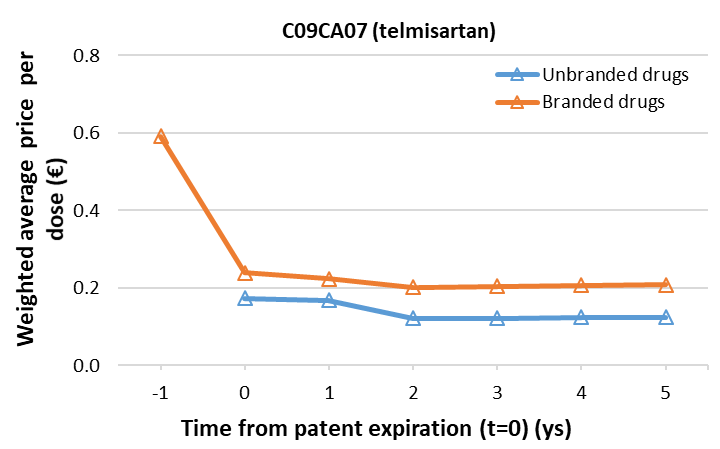

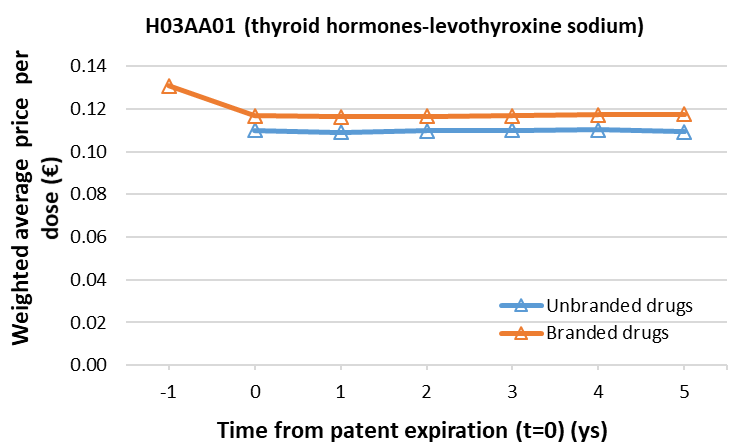


**b) year of patent expiration = 2014**


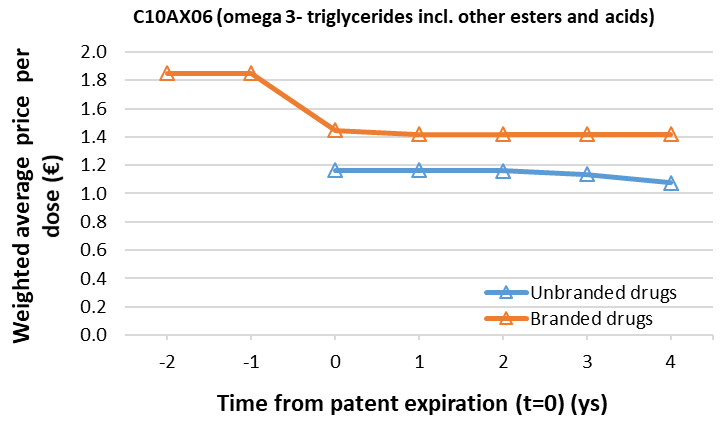

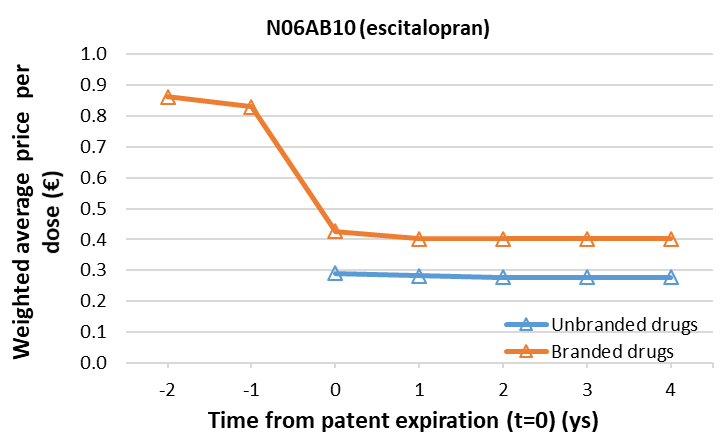


**c) year of patent expiration = 2015**


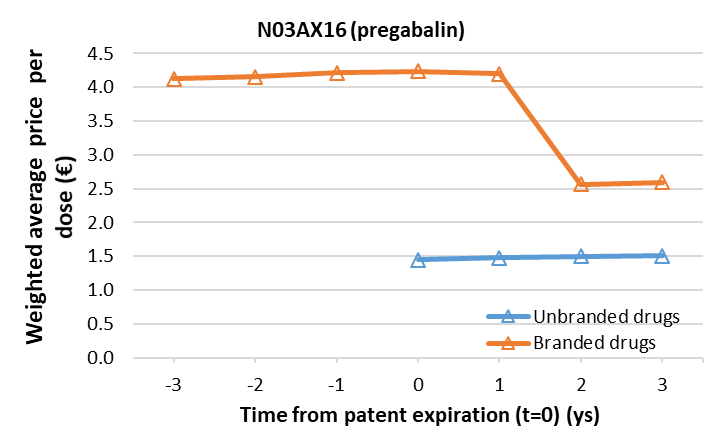

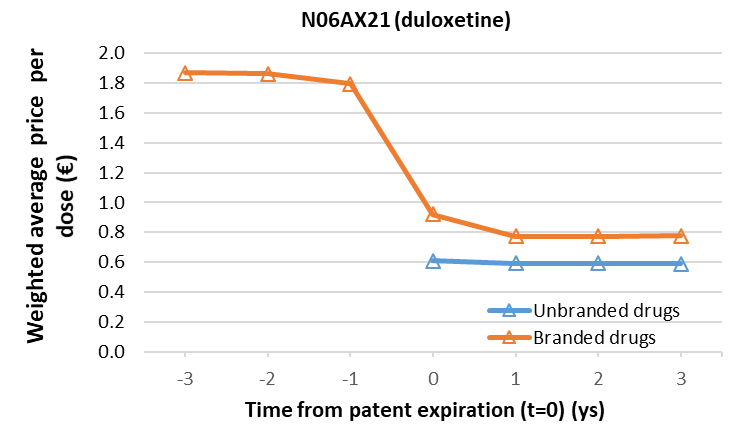


**d) year of patent expiration = 2016**


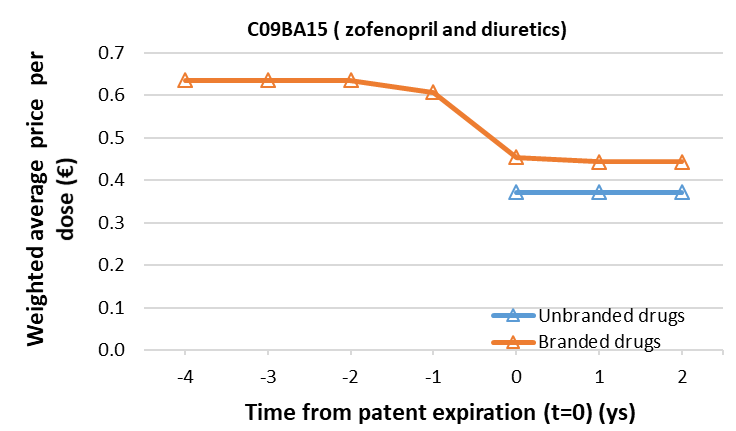

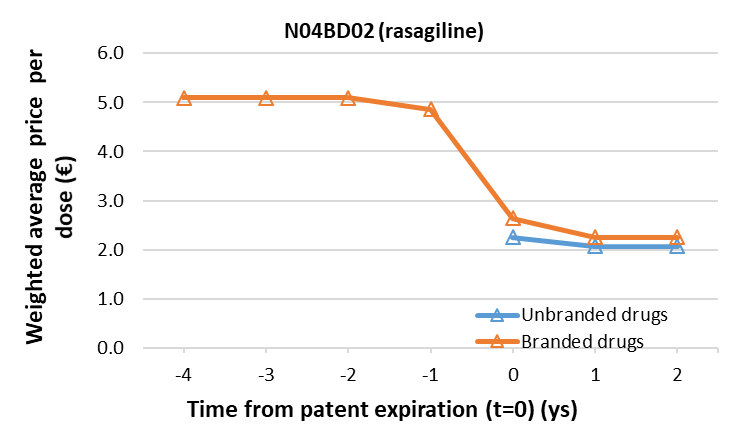

Supplement: Supplementary file 1 [file Data_Sheet_1.docx]
